# Repeated use of rich pictures to explore changes in subjective experiences over time of patients with advanced cancer

**DOI:** 10.1002/cnr2.1428

**Published:** 2021-07-26

**Authors:** Zarah M. Bood, Michael Scherer‐Rath, Mirjam A. G. Sprangers, Liesbeth Timmermans, Ellen van Wolde, Sayra M. Cristancho, Fenna Heyning, Silvia Russel, Hanneke W. M. van Laarhoven, Esther Helmich

**Affiliations:** ^1^ Department of Medical Oncology, Cancer Center Amsterdam Amsterdam University Medical Centers, University of Amsterdam Amsterdam The Netherlands; ^2^ Faculty of Philosophy, Theology, and Religious Studies Radboud University–Nijmegen Nijmegen The Netherlands; ^3^ Department of Medical Psychology Amsterdam University Medical Centers, University of Amsterdam Amsterdam The Netherlands; ^4^ Department of Primary and Community Care Radboud University Medical Centre Nijmegen The Netherlands; ^5^ Centre for Education Research & Innovation and Department of Surgery, Health Sciences Addition, Schulich School of Medicine and Dentistry Western University London Ontario Canada; ^6^ Association of Topclinical hospitals STZ Utrecht The Netherlands; ^7^ Independent Artist Amsterdam The Netherlands; ^8^ Center for Education Development and Research in Health Professions University Medical Center Groningen, University of Groningen Groningen The Netherlands

**Keywords:** cancer, metastasis, methods, patients

## Abstract

**Background:**

The combination of verbal and visual tools may help unravel the experiences of advanced cancer patients. However, most previous studies have focused on a specific symptom, at only one moment in time. We recently found that a specific visual tool, originating from systems thinking, that is, rich pictures (RPs), could provide a more comprehensive view of the experiences of patients with advanced cancer.

**Aims:**

To examine whether the repeated use of RPs can make changes in subjective experiences of patients living with advanced cancer visible over time.

**Methods and results:**

We performed a prospective study with a generic qualitative approach that was mostly informed by the process of grounded theory. We invited patients to make an RP twice, at the start of the study, and again after 2 months. Both RP drawing sessions were directly followed by a semi‐structured interview. Patients with all types of solid tumors, above the age of 18, and with a diagnosis of advanced, incurable cancer, were eligible. Eighteen patients participated and 15 patients were able to draw an RP twice. In eight RP‐sets, considerable differences between the first and second RP were noticeable. Two patterns were distinguished: (1) a change (decline or improvement) in physical health (five patients), and/or (2) a change in the way patients related to cancer (three patients).

**Conclusion:**

RPs are a valuable qualitative research method that can be used to explore the experiences of patients with advanced cancer, not only at a single point in time but also over time.

## 1 BACKGROUND

Having advanced incurable cancer has a profound impact on the quality of life of cancer patients.^1^, ^2^, ^3^, ^4^ Patients may not always be able to find words to express what they feel, particularly in such confusing and distressing circumstances.^5^, ^6^ Hence, current methods to capture subjective experience, such as questionnaires and in‐depth interviews, possibly only uncover part of the experiences of patients with advanced cancer.^7^, ^8^, ^9^ This is also important because patient's experiences can change over the course of their illness.^10^ For example, a systematic review and meta‐analysis by van Kleef et al. shows that cancer patients experienced an improvement in some physical symptoms and emotional functioning during treatment, but a deterioration in role functioning, fatigue, and negative feelings about hair loss.^11^ If we could invite these patients to tell their story through visuals, in addition to spoken language, we might gain a more complete understanding of what they are experiencing during the whole course of their illness.^6^, ^12^, ^13^, ^14^, ^15^

In previous studies, the combination of verbal and visual tools was found to help unravel the experiences of patients.^15^, ^16^, ^17^, ^18^, ^19^ For instance, reviews by Broadbent et al. and Cheung et al. provide an overview of studies that explored patients' illness perceptions using drawings and conclude that drawings can aid in understanding the illness experience.^18^, ^19^ However, it should be noted that in most of the studies reviewed, the drawing instruction had a narrow focus—asking patients to draw themselves, the illness, the part of their body that is affected by the illness, or how the illness made them feel.^18^, ^19^ In addition, in all but one of these studies, drawings were made at only one moment in time.^15^, ^16^, ^17^, ^18^, ^19^ We recently found that a specific visual tool, originating from systems thinking, that is, rich pictures (RPs), could provide a more comprehensive view of the experiences of patients with advanced cancer.^20^ RPs are drawings that research participants create to express their experiences, including different elements, for example, people, materials, processes, interactions, and feelings.^5^, ^6^, ^12^, ^13^, ^21^

RPs were found to combine different aspects of patients' lives, communicating these in one single snapshot.^20^ This promising finding encouraged us to address the subsequent question: How can the repeated use of RPs make changes in subjective experiences of patients with advanced cancer visible over time and what are the most optimal research conditions to achieve this? We explored these questions in a qualitative study among the same patients with advanced cancer who had drawn an RP previously.^20^

## 2 METHODS

### 2.1 Study design and patients

Reporting of the study was done according to the consolidated criteria for reporting qualitative research. This study adopted a generic qualitative approach that was mostly informed by the process of grounded theory.^22^ We applied purposive sampling and started data analysis alongside data collection to inform subsequent sampling and data collection. We invited patients to make an RP twice, at the start of the study, and again after 2 months. Both RP drawing sessions were directly followed by a semi‐structured interview. The findings of the first round of RP interviews were previously published.^20^ The present study will specifically focus on the repeated use of the RPs.

We adopted a constructivist research paradigm acknowledging that knowledge is co‐created between researchers and patients.^23^ Patients constructed their own reality when making an RP, which was refined during the interaction with the interviewer (Z.M.B.), a female PhD student with a research masters in global (mental) health, who was trained in RP interviewing by E.H., a female elderly care physician and experienced RP researcher with a PhD in qualitative research. The backgrounds of the other researchers (ie, medicine, theology, arts, qualitative and RP research) shaped the interpretation of the RPs further.

Patients were recruited from the Department of Medical Oncology of the Amsterdam University Medical Centers, Location AMC, the Netherlands. Patients with all types of solid tumors, above the age of 18, and with a diagnosis of advanced, incurable cancer, were eligible. They were consecutively approached during appointments at the hospital by their attending oncologist. If they expressed interest in participating in the study, they were contacted by Z.M.B. for further information on the study, including the goals of the study, and to obtain formal informed consent. Patients did not know Z.M.B. before start of the study.

### 2.2 Data collection

During the first phase of the research, we included 18 patients, as this allowed for some loss‐to‐follow‐up enabling a sufficiently large sample size to obtain meaningful findings.^20^, ^23^ Clinical data, such as performance status, were obtained from the medical chart. From February till June 2018, we conducted two RP sessions of approximately 1 hour with each patient, with an interval of 2 months, in a setting of their preference: at the hospital or their own home, and with or without a family member or friend being present. In both sessions, we asked patients to make an RP that depicted how they experienced living with cancer at the moment in time. Patients were encouraged to include all aspects of their life that they felt were relevant to their experience. When the RP was finished, the interviewer invited the patient to explain the content of the drawing, which was followed by more specific questions about the colors, shapes, and specific elements used. All interviews were audio recorded. During the whole study period, Z.M.B. kept a notebook to collect field notes. A detailed description of the procedure of the interviews has been described previously.^20^

#### 2.2.1 Repeated use

To explore different options for instructing patients for drawing the second RP, we divided the patients into three conditions at the time of the second interview. In condition one, patients were asked to make a completely new RP. In condition two, patients were presented with a copy of their RP from the first interview and subsequently asked to make a new RP. We left it to the patient's own discretion whether the new RP would be related to the first, for example, by expanding or deleting elements, or would be unconnected. In condition three, patients were given a copy of their RP from the first interview and invited to make adjustments on the copy itself, rather than making a new RP. For example, patients could adjust elements, erase elements, add new elements, change colors, or even leave the RP as it was when they felt it still represented their current experience.

Patients were assigned to the three conditions alternatingly, in order of appearance at the second interview, such that the first available patient was assigned to condition one, the second patient to condition two, the third to condition three, the fourth to condition one, etc. However, patients could change conditions during the RP session when they were not able or willing to follow the instructions specific to those conditions. For instance, when patients expressed a wish to make a new RP, they were allowed to switch to condition three, or when patients opposed to making a new RP, they could switch to condition two.

### 2.3 Data analysis

Interviews were transcribed verbatim and used to support the RP analysis. Transcripts were not returned to patients for additional comments. Data analysis of the combined “Rich Picture/interview” data included both inductive and deductive approaches. The first set of RPs was analyzed in an inductive way, as described in detail before.^20^ During so‐called “RP viewing sessions” one single RP was analyzed in detail for an hour by six to eight researchers who had experience with RP research, using content analysis.^24^, ^25^ In gallery walks, all RPs were pinned to a wall in random order. Attendees (researchers with backgrounds in medicine, theology, arts, qualitative research, and RP research, and also cancer patients) walked along to study the RPs and subsequently discussed the RPs together, looking for patterns, disparities, and similarities across the entire data set. The RP viewing sessions and gallery walks resulted in an initial set of open codes, that were further developed and clustered into themes through open and focused coding of the corresponding interview transcripts.^20^

The second set of RPs was again analyzed by Z.M.B. and E.H. and, for each patient, compared to the first RP of that particular patient. We explicitly looked for similar or different metaphors, visual motifs, and change of color or size of specific elements between the first and second RP.

To support our analysis of the subjective experiences of patients with advanced cancer, our interpretation of how patients relate to cancer, and whether this changed over time, we adopted a deductive approach, alongside the inductive analysis. Deductive analysis was directed by a theoretical model describing modes of relating to contingent life events developed by Kruizinga et al.^26^ Contingent life events are unexpected life events that conflict with the goals people have in life. The model describes four modes: denying, acknowledging, accepting, and receiving. Denying means that people do not engage in the process of interpretation of the life event, and hence have no existential questions and (deliberately) pretend the event does not affect one's life. In the acknowledging mode, people recognize that the life event has an impact on their life and that life goals are affected. Accepting means that people acknowledge the impact of the life event, and also take the first steps to create a new life story. The last mode, receiving, describes the full integration of the life event in one's life story. We studied the combined “Rich Picture/interview” data to arrive at an interpretation of the mode of relating to contingent life events that might be most applicable for each patient. Interpretations of the data were initially discussed by Z.M.B. and E.H. and subsequently presented to the full research team to reach a final consensus.

### 2.4 Ethical considerations

No formal ethical approval was needed for the study, as confirmed by The Medical Ethics Review Committee of the Academic Medical Centre, since the Medical Research Involving Human Subjects Act is not applicable (ref. no W17_476 # 17.549). We obtained written informed consent from all patients and patients were informed about their right to withdraw from the study at any moment. Patients were informed that their RPs would be used for research purposes and could be displayed to others for data analysis. All patient data were treated confidentially and stored in a protected database in the hospital.

## 3 RESULTS

### 3.1 Patients

Eighteen patients participated in the first session of RP drawing and interviews (Table 1).^20^ Five patients were lost to follow up during the study. Two of them passed away before the second session, one declined further participation, and two could not find time due to busy private schedules.

**TABLE 1:** Patients characteristics (*n* = 18)

Partici‐pant number	Date first interview	Date second interview	Gender	Age	Months between diagnosis^a^ and first interview	WHO performance status (first interview)	WHO performance status (second interview)	Interview condition second interview: original placement	Interview condition second interview: final placement
P1	Feb. 2018	May 2018	Male	66	46	0	0	1	1
P2	Mar. 2018	May 2018	Male	31	27	0	0	2	2
P3	Mar. 2018	May 2018	Female	64	13	0	0	1	3
P4	Mar. 2018	June 2018	Male	65	37	0	0	3	3
P5	Mar. 2018	May 2018	Female	59	2	Unknown	Unknown	3	3
P6	Mar. 2018	May 2018	Male	49	2	1	2	3	3
P7	Mar. 2018	May 2018	Male	45	33	0	1	2	3
P8	Mar. 2018	LTFU^b^	Female	68	4	1	‐	‐	‐
P9	Mar. 2018	LTFU^b^	Female	63	27	2	‐	‐	‐
P10	Mar. 2018	May 2018	Female	56	8	1	3	1	1
P11	Apr. 2018	LTFU^b^	Female	75	19	1	‐	‐	‐
P12	Apr. 2018	LTFU^b^	Female	46	14	1	‐	‐	‐
P13	Apr. 2018	June 2018	Male	79	33	2	1	2	3
P14	Apr. 2018	June 2018	Female	73	7	1	0	1	1
P15	Apr. 2018	LTFU^b^	Female	81	32	1	‐	‐	‐
P16	Apr. 2018	June 2018	Male	71	40	1	1	3	3
P17	Apr. 2018	June 2018	Female	60	4	1	0	2	2
P18	May 2018	June 2018	Female	73	21	0	0	1	1

^a^ Specifically the diagnosis of *advanced* cancer.

^b^ Lost‐to‐follow‐up.

### 3.2 Findings

Five out of 13 RP‐sets (first and second round of RPs) showed little to no difference between the 2 RPs, with the patients explaining that their life indeed had not changed noteworthy. These patients preferred to work on a copy of their first RP (condition three), because it still represented their current situation.

In the other eight RP‐sets, considerable differences between the first and second RP were noticeable. Two patterns could be distinguished: (1) a change (decline or improvement) in physical health (five patients), and/or (2) a change in the way patients related to cancer (three patients). Although represented across all three interview conditions, patients whose physical and/or mental condition considerably changed more often preferred to make a new RP (condition one or two). Two patients expressing considerable change, however, were assigned to condition 3 and were able to show changes by building on/changing their previous RP. Below we will describe the results of five patients as an example.

#### 3.2.1 RPs illustrating changes in physical health

**FIGURE 1:**
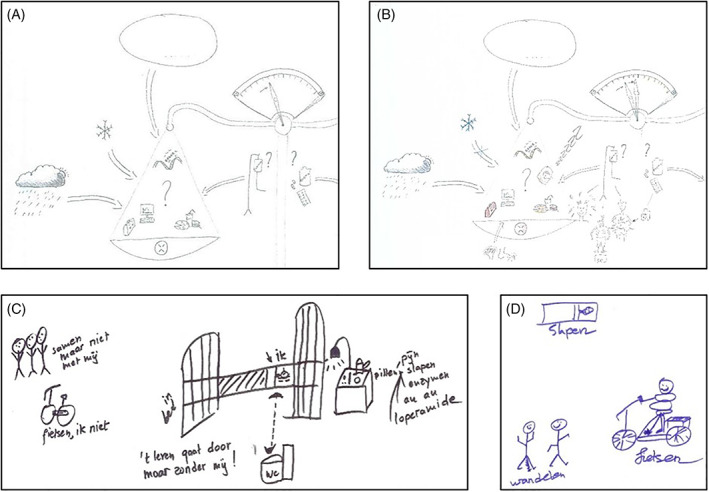
Rich pictures (RPs) that make differences in physical health visible. (A) Part of first RP of patient P6.* (B) Part of second RP of patient P6. (C) Part of first RP of patient P14.** (D) Part of second RP of patient P14.*** *The complete RP was published before by Cancer Medicine, 2019 Sep; 8 (11): 4957‐4966. **Translations (from left to right): together but not with me; cycling, not me; in bed; life goes on without me!; me (“ik”); toilet (“WC”); pills—pain, sleeping, enzymes, ouch ouch, loperamide. ***Translations: slapen = sleeping; wandelen = going for a walk; fietsen = cycling

A decline in physical health was visible when comparing the first and second RP of patient P6 (Figure 1(A,B)). In his first RP, this patient drew a snowflake to represent the neuropathy he experienced when the weather was cold. He also drew an empty balloon symbolizing the emptiness of his days due to his inability to work. Whereas he put a cross through the snowflake in his second RP, as the weather got better, he added a drawing of his hand and feet hurting because of neuropathy (Figure 1(B)). He also added a drawing of himself sleeping on a pillow, having stomach pains, with his ribs showing through his skin, and his hair falling out. In sum, he was more tired and in pain, and suffered from weight loss and alopecia due to chemotherapy. This visualized decline was confirmed by his medical record that indicated an elevated WHO performance status (from one to two), indicating deteriorating physical health.

The RPs of patient P14 showed improved physical health. In the first RP, the central element was the patient lying in bed, in close proximity of the toilet and medication, whereas in the second RP only a small bed was drawn (Figure 1(C,D)). In addition, in the first RP, the patient specifically wrote down that she could not cycle anymore and could not join others in activities. In the second RP, she drew pictures of cycling and hiking. The increasing physical health of the patient was in line with the improvement of her WHO performance status (one to zero).

#### 3.2.2 RPs illustrating differences in the way patients relate to cancer

**FIGURE 2:**
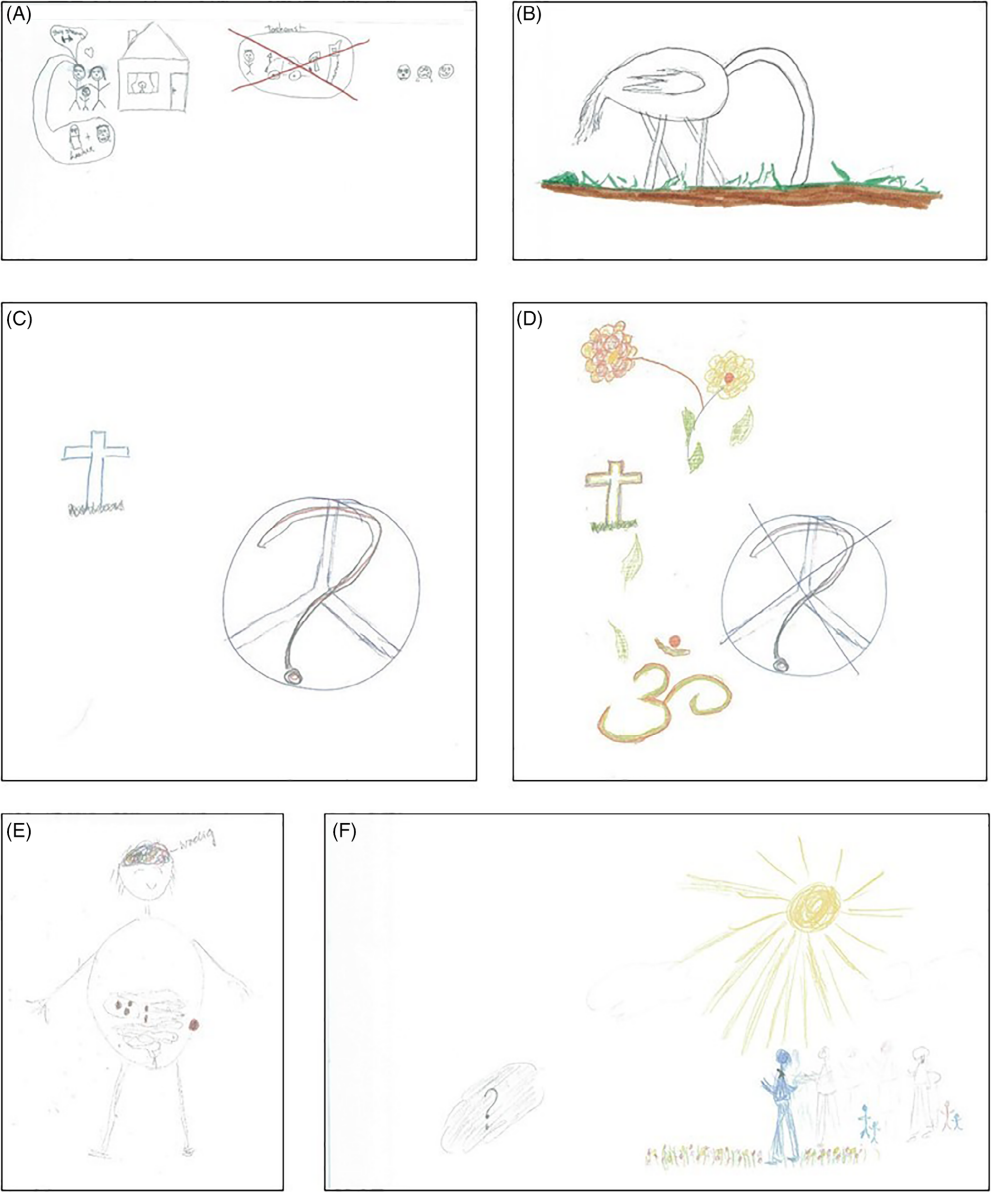
Rich pictures (RPs) that make differences in in the way patients relate to the cancer visible. (A)* First RP of patient P2. (B) Second RP of patient P2. (C) Part of first RP of patient P5. (D) Part of second RP of patient P5. (E) First RP of patient P18. (F) Second RP of patient P18. *The RP was published before by Cancer Medicine, 2019 Sep; 8 (11): 4957‐4966

The repeated use of RPs allowed us to identify distinct ways of relating to cancer over time. Patient P2 drew himself in his first RP thinking about death and the loss of his future, and explicitly depicted the sadness and anger this invoked (Figure 2(A)). He acknowledged he had cancer, but did not accept it. For his second RP, however, he just drew one element: an ostrich sticking its head into the sand (Figure 2(B)). He explained: “I bury my head in the sand, I just pretend it is not there.” In other words, after initially acknowledging cancer, he now decided to try to deny the existence of cancer.

A change from acknowledging to acceptance was found in patient P5. In her first RP, she depicted great sadness and anger about the loss of her life and drew, among other things, her grave and a peace sign with a question mark over it (Figure 2(C)). She had added the latter sign because the question “is this supposed to bring me peace?” kept popping up in her head. Hence, this patient acknowledged her situation, but steps toward acceptance were not yet taken. In her second RP, she put a cross through the peace sign and the question mark and colored the grave with orange and green, because her immanent death was less haunting. She also added three elements to the RP: flowers, leaves, and an Ohm‐sign (Figure 2(D)). These, respectively, represented the happiness about being able to exercise her hobbies, the inner peace she had now found, and the regained connection with her religion. About finding inner peace she said: “I think the acceptance is just way bigger. A certain calmness, more inner peace than the first time.”

Patient P18 showed a transition from acknowledging to receiving. In her first RP, she depicted the cancer in her body and the mental struggles she was having (Figure 2(E)). She acknowledged the cancer and the effect it had on her life. In her second RP, she drew herself walking in the sun, on flowers, towards the unknown future (Figure 2(F)). As she explained, the cancer is her shadow, living with her, and death is a little bird sitting on her shoulder. The sun and flowers were drawn because she is enjoying life and because she felt like she had become a happier person. She explained that she actually gained a lot from the cancer: “I think that what you go through, if you don't let it get you down, can give you a lot of strength and energy, and also happiness. […] So I am actually happy with the cancer, as weird as it sounds.”

## 4 DISCUSSION

### 4.1 Main findings

Our results suggest that the repeated use of RPs can make changes in the subjective experience of living with cancer visible over time, specifically by giving insight into changes in physical health and the way patients related to living with cancer (denying, acknowledging, accepting, or receiving^26^). In addition, our study suggests that for repeated use of RPs different interview formats yield similar results, that is, inviting patients to make a new drawing vs providing them with a copy of their previous RP to work on.

### 4.2 What this study adds

To the best of our knowledge, this was the first study that examined the potential of RPs as a method for capturing changes in the subjective experience of living with cancer over time. Previous research has applied RPs to gain insight into subjective experiences at one time only.^20^ Since we found that the repeated use of RPs revealed meaningful changes over time, we suggest that the repeated use of RPs has potential as a longitudinal follow‐up tool or as an outcome measurement before and after an intervention, for example, a mental health intervention for advanced cancer patients.

In comparison to other studies that used drawings to explore patient experience,^16^, ^18^, ^19^ ^,^, the added value of RPs lies in the ability to represent all important themes of a patient's life in one single snapshot. When making a RP, patients are invited to include everything that they consider relevant to the situation in their drawing, from events, people, and objects to feelings and emotions, instead of just focusing on one element such as pain or anxiety.^16^, ^18^, ^19^ Hence, the repeated use of RPs allows for insight in changes in all aspects of a patient's life. Furthermore, previous research such as Van Kleef et al.^11^ has shown that even though the overall quality of life (QoL) of cancer patients seems to remain stable over time, changes in different health‐related QoL domains are often present and not always captured by the QoL score. This suggests that a measurement method, such as an RP, that gives insight in these different domains, could be of added value. RPs could not only uncover those specific changes in QoL domains, but also give insight into the relationships between these QoL domains.

A next step in RP research could be to investigate the use of RPs in combination with other measurement methods, such as questionnaires. A relevant question will be whether RPs in combination with questionnaires would provide a more comprehensive representation of the experiences of cancer patients than either one alone. In this way, the effect of interventions could possibly be measured more comprehensively.

### 4.3 Strengths and limitations

A strength of our study is that it explored the repeated use of RPs in a rigorous way. We built upon previous RP research, examined three different interview conditions, and performed an interim analysis. The interim analysis allowed us to make the best possible use of our 13 patients, as we did not waste interviews on interview conditions that were not working. It should be noted that we did not intend to make a formal comparison in terms of outcome between the three modes of instruction, but rather aimed to investigate what approach would work in clinical practice. Patients who experienced little to no changes in their illness experience, preferred receiving a copy of their previous RP instead of making a new RP. In turn, some patients had a clear preference for making a new RP, because the first RP did not reflect their current experience. Based on these findings, we argue that when patient preferences for the drawing of RPs is taken into account, repeated use of this interview format is feasible.

Next, researchers from different backgrounds, but also patients, an artist, and doctors, gave us the opportunity to incorporate many backgrounds in the data interpretation, enriching the results. In this study, we aimed for consensus in data interpretation between the different actors, while the level of agreement between coders was not of main interest. A final strength of this study is the use of the theoretical model of Kruizinga et al.^26^ to support the interpretation of how patients relate to the cancer at the two moments of time.

Generalizability of our findings, however, might be limited due to practical factors. RPs may not be suitable for all patients with advanced cancer, as engaging in an RP session of approximately an hour may be too burdensome. In addition, not all patients may feel comfortable with expressing themselves using visual methods. This may be patient, age, and culture dependent. Finally, as is the case with almost all measurements, drawing of an RP may by itself induce a change in one's experience of living with cancer, because a reflective process is brought into motion. The sensitivity of RPs to pick up changes after an intervention compared to a control group needs further investigation.

#### Conclusions

RPs were found to provide insight into changes in physical condition as well as how patients relate to living with cancer. RPs are a valuable qualitative research method that can be used to explore the experiences of patients with advanced cancer, not only at a single point in time but also over time.

## CONFLICT OF INTEREST

The authors declare that there is no conflict of interest.

## AUTHOR CONTRIBUTIONS

All authors had full access to the data in the study and take responsibility for the integrity of the data and the accuracy of the data analysis. *Conceptualization*, H.W.M.L. and E.H.; *Methodology*, H.W.M.L., E.H., Z.M.B.; *Investigation*, Z.M.B.; *Formal analysis*, all authors; *Writing‐original draft*, Z.M.B.; *Writing‐review & editing*, all authors; *Visualization*, Z.M.B.; *Supervision*, H.W.M.L.; *Funding acquisition*, H.W.M.L.

## ETHICS STATEMENT

No formal ethical approval was needed for the study, as confirmed by The Medical Ethics Review Committee of the Academic Medical Centre, since the Medical Research Involving Human Subjects Act is not applicable (ref. no W17_476 # 17.549). Written informed consent was obtained from all patients.

## Data Availability

The datasets used and/or analyzed during the current study are available from the corresponding author on reasonable request.

## References

[1] Breitbart W , Rosenfeld B , Pessin H , et al. Depression, hopelessness, and desire for hastened death in terminally ill patients with cancer. J Am Med Assoc. 2000;284:2907‐2911. 10.1001/jama.284.22.2907 11147988

[2] Hartog I , Scherer‐Rath M , Kruizinga R , et al. Narrative meaning making and integration: toward a better understanding of the way falling ill influences quality of life. J Health Psychol. 2017;25:1‐17. 10.1177/1359105317731823. PMC7221864 28948830

[3] Smith EM , Gomm SA , Dickens CM . Assessing the independent contribution to quality of life from anxiety and depression in patients with advanced cancer. Palliat Med. 2003;17:509‐513. 10.1191/0269216303pm781oa. 14526884

[4] Jordhoy MS , Fayers P , Loge JH , et al. Quality of life in advanced cancer patients: the impact of sociodemographic and medical characteristics. Br J Cancer. 2001;85:1478‐1485. 10.1054/bjoc.2001.2116. 11720432 PMC2363932

[5] Bell S , Morse S . How people use rich pictures to help them think and act. Syst Pract Action Res. 2013;26:331‐348.

[6] Cristancho S , Bidinosti S , Lingard L , Novick R , Ott M , Forbes T . Seeing in different ways: introducing "rich pictures" in the study of expert judgment. Qual Health Res. 2015;25:713‐725. 25281244 10.1177/1049732314553594 PMC5578751

[7] Kruizinga R , Hartog ID , Jacobs M , et al. The effect of spiritual interventions addressing existential themes using a narrative approach on quality of life of cancer patients: a systematic review and meta‐analysis. Psychooncology. 2016;25:253‐265. 10.1002/pon.3910. 26257308

[8] Pearce MJ , Coan AD , Herndon JE 2nd , et al. Unmet spiritual care needs impact emotional and spiritual well‐being in advanced cancer patients. Support Care Cancer. 2012;20:2269‐2276. 10.1007/s00520-011-1335-1. 22124529

[9] Anderson WG , Alexander SC , Rodriguez KL , et al. "what concerns me is…" expression of emotion by advanced cancer patients during outpatient visits. Support Care Cancer. 2008;16:803‐811. 10.1007/s00520-007-0350-8. 17960430 PMC2838893

[10] Al‐Batran SE , Ajani JA . Impact of chemotherapy on quality of life in patients with metastatic esophagogastric cancer. Cancer. 2010;116:2511‐2518. 10.1002/cncr.25064. 20301114

[11] van Kleef JJ , Ter Veer E , van den Boorn HG , et al. Quality of life during palliative systemic therapy for Esophagogastric cancer: systematic review and meta‐analysis. J Natl Cancer Inst. 2020;112:12‐29. 10.1093/jnci/djz133. 31251346

[12] Cristancho S . Eye opener: exploring complexity using rich pictures. Perspect Med Educ. 2015;4:138‐141. 10.1007/s40037-015-0187-7. 25947652 PMC4456463

[13] Helmich E , Diachun L , Joseph R , et al. 'Oh my god, I can't handle this!': trainees' emotional responses to complex situations. Med Educ. 2018;52:206‐215. 10.1111/medu.13472. 29044675

[14] Ellis J , Hetherington R , Lovell M , et al. Draw me a picture, tell me a story: evoking memory and supporting analysis through pre‐interview drawing activities. Am J Eng Res. 2013;58:488‐508.

[15] Williams IC . Graphic medicine: comics as medical narrative. Med Humanit. 2012;38:21‐27. 10.1136/medhum-2011-010093. 22282425

[16] Elkis‐Abuhoff D , Gaydos M , Goldblatt R , Chen M , Rose S . Mandala drawings as an assessment tool for women with breast cancer. Arts Psychother. 2009;36:231‐238.

[17] Nainis N , Paice JA , Ratner J , et al. Relieving symptoms in cancer: innovative use of art therapy. J Pain Symptom Manag. 2006;31:162‐169. 10.1016/j.jpainsymman.2005.07.006. 16488349

[18] Broadbent E , Schoones JW , Tiemensma J , et al. A systematic review of patients' drawing of illness: implications for research using the common sense model. Health Psychol Rev. 2019;13:406‐426. 10.1080/17437199.2018.1558088. 30558520

[19] Cheung MM , Saini B , Smith L . Using drawings to explore patients' perceptions of their illness: a scoping review. J Multidiscip Healthc. 2016;9:631‐646. 10.2147/JMDH.S120300. 27920550 PMC5125999

[20] Bood ZM , Scherer‐Rath M , Sprangers MAG , et al. Living with advanced cancer: rich pictures as a means for health care providers to explore the experiences of advanced cancer patients. Cancer Med. 2019;8:4957‐4966. 10.1002/cam4.2342. 31278862 PMC6718737

[21] Armson R . Drawing rich pictures. In: Armson R , ed. Growing Wings on the Way: Systems Thinking for Messy Situations. Axminster, UK: Triarchy Press; 2011:57‐59.

[22] Kahlke RM . Generic qualitative approaches: pitfalls and benefits of methodological mixology. Int J Qual Meth. 2014;13:37‐52. 10.1177/160940691401300119.

[23] Charmaz K . Constructing Grounded Theory. 2nd ed. London: Sage; 2014.

[24] Bell S , Berg T , Morse S . Rich pictures: sustainable development and stakeholders—the benefits of content analysis. Sustain Dev. 2015;24:136‐148.

[25] Berg T , Pooley R . Contemporary iconography for rich picture construction. Syst Res Behav Sci. 2012;30:31‐42.

[26] Kruizinga R , Hartog ID , Scherer‐Rath M , Schilderman H , van Laarhoven H . Modes of relating to contingency: an exploration of experiences in advanced cancer patients. Palliat Support Care. 2017;15:444‐453. 10.1017/S1478951516000936. 27995821

